# Social capital and educational justice as predictors of civic engagement in higher education: the mediating roles of institutional trust and student empowerment

**DOI:** 10.3389/fsoc.2026.1869260

**Published:** 2026-07-09

**Authors:** Marian Mae S. Sapanta, Romel C. Mutya

**Affiliations:** 1 College of Education, Arts and Sciences, Cebu Technological University-Danao Campus, Danao, Cebu, Philippines; 2 Graduate School, Cebu Technological University-Danao Campus, Danao, Cebu, Philippines

**Keywords:** civic engagement, educational justice, higher education, institutional trust, social capital, student empowerment

## Abstract

Aligning with the Philippine Commission on Higher Education’s mission to produce socially responsible and civically active graduates, this study examined college students’ academic experiences in relation to social capital, educational justice, student empowerment, institutional trust, and civic engagement. A survey was conducted among 1,026 college students from selected state universities in Northern Cebu, Philippines. Descriptive and inferential statistics were employed to examine the relationships among the identified constructs. Partial Least Squares Structural Equation Modeling (PLS-SEM) was used to assess the direct and indirect mediating effects of student empowerment and institutional trust on civic engagement. The findings revealed acceptable levels of agreement across all constructs, with institutional trust obtaining the highest mean score (*M* = 4.16). Student empowerment demonstrated the strongest positive influence on civic engagement (*β* = 0.477, *t* = 11.691, *p* < 0.001) and emerged as the most influential mediator, with indirect effects of *β* = 0.199 and *β* = 0.197. The results further indicated that social capital and educational justice exert significant indirect effects on civic engagement through the mediating roles of student empowerment and institutional trust. The findings suggest that fostering an inclusive, equitable, and supportive academic environment plays a vital role in strengthening civic attitudes, responsibility, and active citizenship among college students. The study recommends that higher education institutions sustain civic engagement by implementing practical, service-oriented student programs and community initiatives that promote student empowerment and institutional trust.

## 1 Introduction

In recent years, higher education has expanded its mission beyond instruction and research to include community outreach and other civic-oriented activities. Globally, colleges and universities are committed to producing skilled professionals and nurturing individuals to be socially responsible, critically aware, and civically engaged citizens (Rodríguez-Zurita et al., 2025). Fostering civic engagement among college students in a time characterized by political polarization, declining institutional trust, and youth disengagement is important (Blevins et al., 2021). Civic education has long emphasized knowledge of democratic processes, yet it has become glaringly clear that civic engagement must be informed by and oriented upon larger educational experiences inaccessibility, interpersonal inclusiveness, empowerment, and social networks (Elliott, 2018).

Higher education institutions (HEIs) have assumed a significant role in preparing learners with the skills, competencies, values, and motivation needed for civic participation. There has been growing recognition that higher education institutions are avenues for academic development and critical environments for cultivating civic behavior and civic responsibility (Kepaliene et al., 2023). Moreover, social capital and educational justice are emerging key predictors that translate how institutional frameworks transform into civic outcomes (Aramburuzabala et al., 2024). Both have contributed to how students understand their roles in the institution and their capabilities to act in the public interest. While, social capital is broadly defined as the value we derive from our relationships and connections we choose to have. This also describes the norms, networks, and trust that promote collective action and collaboration (Satiani and Hadiwijoyo, 2024). To some studies, HEIs’ can effectively develop civically aware and engaged students if they can facilitate peer interactions, institutional trust, and faculty involvement religiously (Trolian and Parker, 2020).

On the other hand, educational justice refers to equal opportunities for students to succeed, which is globally recognized as a key driver of inclusive civic engagement. It is believed that, when students feel empowered in a fair academic setting, they are more likely to experience and develop a sense of civic duty that motivates them to make a difference both personal and academic (Franch, 2020). Drawing from Putnam’s social capital theory, student empowerment functioned as a critical mediator that increases civic skills among individuals. At the same time, institutional trust also acts the same which is grounded in perceptions of fairness and legitimacy aligned with Rawl’s theory of justice. Evidently, in Philippine Higher Education, there has been an increasing emphasis on students’ active participation in nation-building, especially in light of the issues and challenges posed by disinformation, political disenfranchisement, and unequal access to education. In response, the Universal Access to Quality Tertiary Education Act, Republic Act No. 10931 is an initiative of the Philippine government which aimed to promote civic participation and social mobility in various educational settings and programs. This venture benefitted the underprivileged members of society. However, factors such as social inequality and perceived institutional neglect (Ablian et al., 2023) undermine the ability of underrepresented groups in the community to stay academically aligned with civic duties.

In a world where youth doubt their political views and limit their freedom of expression, an academic institution’s credibility and responsiveness play a vital role in student know-how of their civic rights and duties. From then on, the fullest confidence from and trust in institutions allows for more participation from students when they perceive their voices as significant (Schneider, 2022). By contrast, disengagement, complacency and even resistance can occur without these factors. These issues and problems are especially prevalent in state universities and colleges (SUCs) in Philippines where there is the limited availability of resources, poor governance, as well as regional disparities and inequality (Moreno and Sulasula, 2023). Additionally, students from rural and low-income communities lack access to leadership programs, mentors, decision-making bodies and other civic- aligned opportunities. Despite these obstacles, many local institutions serve as important platforms for cultivating civic participation through student organizations, community engagement, and academic service-learning (Palid et al., 2023). With these concerns, the primary gap is determining which institutional roles and socio-cultural aspects best foster civic engagement, and how they can work together effectively.

Thus, this study explores the roles of social capital, educational justice, institutional trust and student empowerment on civic engagement. From the perspective of this study, social capital refers to student’s connections, relationships and support systems while educational justice denotes student’s experiences on fairness and equal access to resources within the academic setting. Further, the level of trust, responsiveness and transparency attributes student’s institutional trust meanwhile student empowerment reflects their active participation and how they influence their academic experiences. Henceforth, civic engagement can be described as student’s active engagement in academic activities, community- oriented and socially responsible initiatives that promote collective welfare and democratic participation.

With these significant constructs, this study examines how social capital and educational justice function as predictors of civic engagement among students in higher education institutions (HEIs) in Cebu, Philippines. This research also looked into how institutional trust and student empowerment mediate this relationship. Specifically, it investigates the interplay between structural factors and psychological mechanisms, highlighting institutional trust and student empowerment as mediating factors in the interaction hypothesized by the study. Hence, the study employs a comprehensive and multidimensional framework to analyze civic behavior in education. Furthermore, the study is significant to students of higher education in Cebu, Philippines’ educational landscape which includes state colleges and universities students who have been encouraged and enjoined to actively participate in nation building or most essentially, regarding the emerging problems like political disinformation, marginalization as well as inequitable resource distribution. The findings can provide practical insights to policymakers, leaders and civic organizations that will inform how they design and develop initiatives to foster student engagement in all aspects of learning—as students and as active, responsible citizens.

### 1.1 Hypothesis development and literature review

#### 1.1.1 Social capital, institutional trust, and student empowerment

Putnam’s Theory of Social Capital posits that social capital is defined as social networks that allow individuals to work together for their own benefit, trust one another, and give back to others. According to Neves et al. (2019), these bonds made it easier and more convenient to share important information and relevant resources, as well as provide emotional support. If students get along with each other, they are likely to do better and graduate (Morrison, 2021). In the study by Okpych and Gray (2021), it was also explained that social capital, through bonding (strong ties) and bridging (connections between groups), enable students to perform better and excel in school. When kids have bonding capital, they feel a sense of belonging and are protected. Sormani and Rossano-Rivero (2023) stated that students who are part of diverse and unified social networks exhibit better positive social behaviors and are more civically involved in their surroundings. This will exemplify that social capital encourages democratic ideology of learning institutions which strengthens the student engagement in learning environments. This finding was consistent with Huang (2025) where social capital driven by institutional trust is a key predictor of student satisfaction and retention across students from both less-privileged as well as privileged backgrounds. Truly, social capital can help students navigate challenging times and serve as a strong support system both at home and at school. Thus, it is hypothesized that:

*H1 and H2: Social capital positively influences institutional trust and student empowerment*

#### 1.1.2 Social capital and civic engagement

Dee (2020) affirmed that when young adults are able to actively participate in diverse social environments and networks profoundly influences their civic development. Building places that nurture social capital, such as peer mentoring or assistance, learning hubs, and student club groups, increases the likelihood that graduates will become productive citizens who care deeply about their local and global communities. Lopez (2020) reveals that digital learning communities on various online platforms can also help build and develop bridging and bonding capital, particularly in hybrid and online learning settings. Other researchers argue that not all students have equal access to digital social capital, particularly those from disadvantaged backgrounds. With that, schools and other groups must ensure that everyone can exchange ideas and share opinions with both online or offline interactions (Milenkova and Lendzhova, 2021). Ultimately, everyone will enjoy school more and find it easier to attend and graduate. Similarly, SDG 4 of the United Nations states that everyone should have access to quality education and continuous learning throughout their lifetime. Thus, it is hypothesized that:

*H3: Social capital has a direct positive effect on civic engagement.*

#### 1.1.3 Educational justice, institutional trust, student empowerment, and civic engagement

As Rawls’s theory of justice suggests that the general principles of equity and equality must retain its significance. Recent debates have concerned how schools must need to identify and tear down systemic bars and impediments, and distribute resources equitably. Burke and Lumb (2024) pointed out that educational justice involves equitable access to the same resources, voice for everyone, an embrace of diversities and a redistribution of power despite differences in societal status. Additionally, Gube and Arat (2024) emphasized the importance of institutional policy in maintaining justice within higher education institutions. Their research shows that the effect on student’s interest in school of promoting diversity, equity and inclusion can be huge. For education to truly practice fairness, significant institutional leaders must also be critically aware and reflect on what is happening in them within their institutions. For instance, students’ willingness and motivation to learn, and their belief in their school strengthen when they perceive their learning environments as fair, inviting, and welcoming. Thus, it is hypothesized that:

*H4, H5, and H6: Educational justice positively influences institutional trust, student empowerment, and civic engagement.*

#### 1.1.4 Institutional trust and civic engagement

In the study by Toquero and Ramos (2024), students enrolled in Philippine state colleges and universities who trusted their school leaders were more likely to perform better academically and demonstrate strong dedication to their institution. This suggests that trust makes people more open to learning new things which helps them understand what the institution aims to achieve in their development and success (Hoque, 2025). Plausibly, students are more participative in school events and other activities if they believe the people in charge care about and think of them. According to Lo (2025), the level of trust students have in their institution influences how they perceive the leaders and their level of involvement in the process. Some other studies have demonstrated the importance of trust in engaging young people and increasing their involvement in civic issues. Accordingly, Philippine state institutions have observed similar trends and situations in which administrators who are trustworthy enough to manage schools are effective in encouraging students to support educational reforms and outreach projects (Borhan, 2025). Thus, it is hypothesized that:

*H7: Institutional trust positively influences civic engagement.*

#### 1.1.5 Student empowerment and civic engagement

Another significant factor in civic engagement is student empowerment, as discussed by Chen and Liu (2020). To empower students, there is a need to give them real involvement, responsibility, and control over decisions which can directly affect their learning in both academic and real-world settings. Zimmerman’s empowerment concepts distinguishes between empowering procedures, such as opportunities and institutional mechanisms. These facilitate collaboration, and empowerment outcomes, including enhanced self-efficacy and exemplary civic values among students (Hofverberg and Sigurdson, 2023). Indeed, Er et al. (2021) found that students who comprehend, utilize, and engage with feedback within their institutions can establish feedback channels. As a result, can foster open dialogue that are more likely to practice and exhibit self-regulation, thereby enhancing courses and programs. Thus, it is hypothesized that:

*H8: Student empowerment positively influences civic engagement.*

#### 1.1.6 Institutional trust mediates the relationship between social capital and civic engagement, and between educational justice and civic engagement

Trust in institutions is essential in connecting social resources to civic behavior. Accordingly, civic participation can include activities such as collaborating with institutions, providing strong support, and striving for social change in the community (Kiss et al., 2022). At this point, these goals make it clear that education is not just about passing on or transferring knowledge it is also about making individuals socially accountable and responsible. Therefore, individuals who actively participate in democratic processes are more likely to trust their institution (Peruzzotti, 2025). Freire (1970) affirms that critical pedagogy significantly impacts the theoretical foundations and underpinnings of civic participation. It asserts that education must be transformative, which empowers students to question and critically examine social structures and systems, thereby equipping them to become active agents of change. Moreover, educational justice means providing all students with what they deserve, regardless of their origin or family background, the same opportunities, resources, and decision-making power in education. In higher education, educational justice is characterized by equitable representation in governance, transparent policy implementation, and a focus on students’ needs (Fatemi, 2025). Several studies have also shown that students are more likely to engage in civic activities and activism when they believe educational practices are fair and trustworthy (Chan, 2023). For instance, Robinson (2024) found, using cross-national data, that institutional trust, enhanced by accountability and communication from leaders, was a strong predictor of academic involvement. The findings highlighted willingness and readiness to take on governance and advocacy tasks and responsibilities. In the study of Lansing et al. (2023) found that trust in university’s administration significantly predicted students’ willingness to lead or participate in community-based programs, particularly when resource allocation and decision-making transparency were evident. Thus, it is hypothesized that:

*H9 and H10: Institutional trust mediates the relationship between social capital and civic engagement, and between educational justice and civic engagement.*

#### 1.1.7 Student empowerment mediates the relationship between social capital and civic engagement, and between educational justice and civic engagement

In college, social capital manifests in relationships and interactions with peers, in dialogues between students and teachers, and in involvement in student organizations. These factors can affect academic performance, especially civic engagement (Beard, 2021). These unique networks enable people to share information, assist and help one another, and enhance their leadership skills. Doing so, also helps the people involved and the organizations they work for (Ridgeway, 2024). Alshammari et al. (2023) conducted a study demonstrated that students with elevated social capital were more likely to actively engage in peer learning communities and civic organizations, both on and off campus. Strong social networks in higher education can predict participation and engagement (Eda et al., 2025) additionally, this research emphasized that students who have supportive peers and organizational networks are expected to have higher levels of volunteerism, institutional engagement, and student empowerment within their learning environment. With this, it is true then that student empowerment is a psychological and structural gateway that links feelings of justice or trust in institutions to active participation in governance, volunteering, and policy formulation (Siddiqui, 2024). On this note, in higher education this talks about students taking charge of their learning environments, school regulations, and community opportunities (Din et al., 2021). While, Stergiopoulou (2025) found that institutional trust significantly affects the relationship between social capital and civic behavior. This means that students who were part of strong social networks, like peer groups or student club organizations, were more likely to act civically when they trusted their institutions. This illustrates the importance of establishing networks and fostering trust in institutions. Thus, it is hypothesized that:

*H11 and H12: Student empowerment mediates the relationship between social capital and civic engagement, and between educational justice and civic engagement*

**Figure 1:**
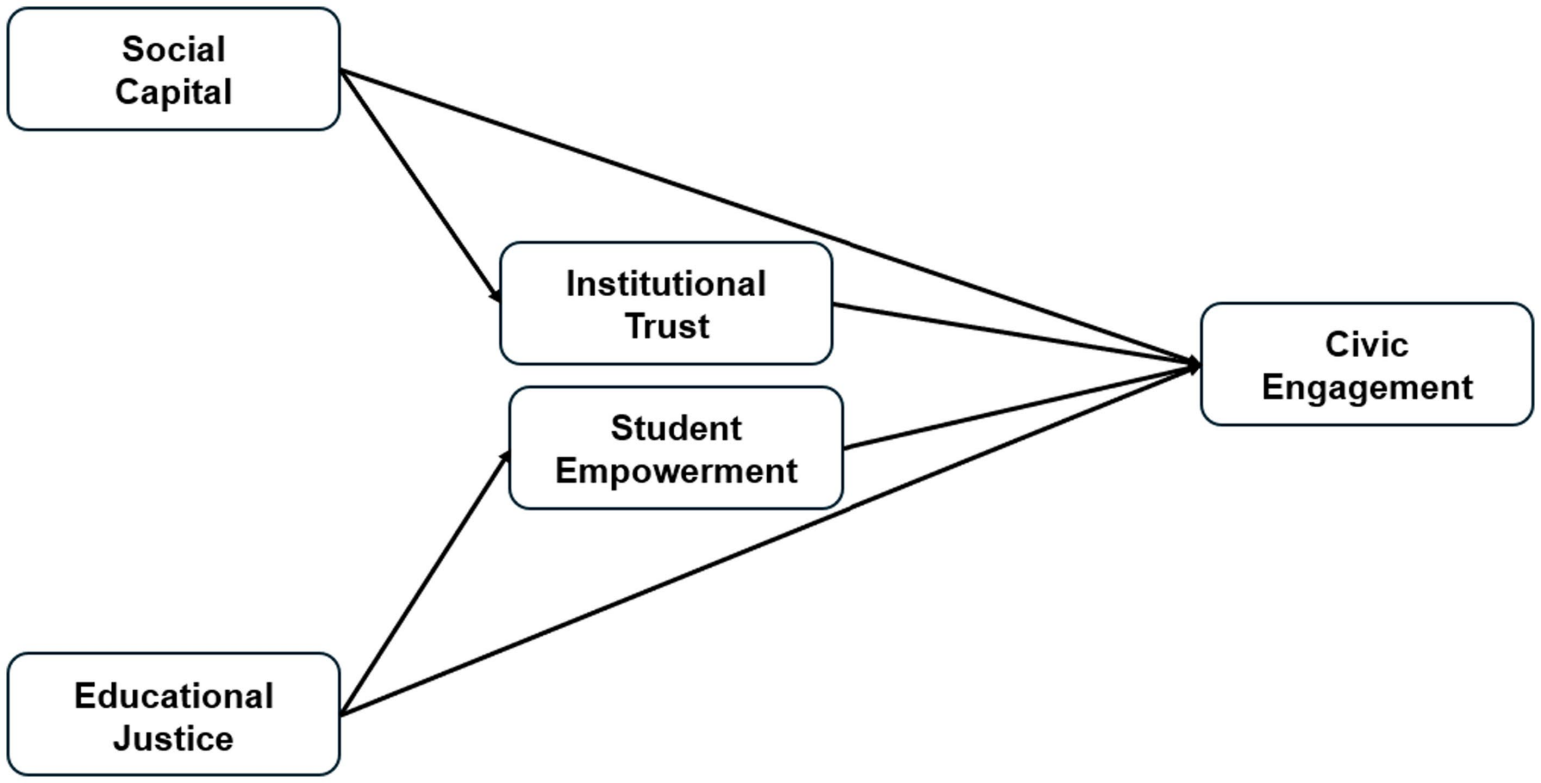
Proposed model of the study.

Figure 1 shows the proposed conceptual model of the study, illustrating the hypothesized relationships among social capital, educational justice, institutional trust, student empowerment, and civic engagement. The model proposes that social capital and educational justice significantly influence institutional trust, student empowerment, and civic engagement. It also hypothesizes that institutional trust and student empowerment mediate the effects of social capital and educational justice on civic engagement.

## 2 Methods

### 2.1 Design

Correlational research design with Partial Least Squares Structural Equation Modeling was employed to examine the predictive relationships between social capital and educational justice, as well as the mediating roles of institutional trust and student empowerment in civic engagement. This assessed and described the degree and direction of association between two or more variables without manipulating them. Partial Least Squares Structural Equation Modeling is a variance-based structural equation modeling technique that primarily analyzes complex interactions between observable and latent variables (Hair et al., 2021). This design is suitable for assessing multiple interdependent relationships simultaneously and examines both direct and indirect (mediated) effects, which could provide a robust predictive framework.

### 2.2 Respondents

The respondents of the study were undergraduate students from selected state universities in Northern Cebu, Philippines where students generally benefit from government-funded free tuition through the implementation of the Universal Access to Quality Tertiary Education Act. The target population are college students from different academic programs and colleges across the selected state universities from the three campuses of Cebu Technological University namely Carmen Campus, Consolascion Campus and Danao Campus. These institutions are characterized by diverse socioeconomic backgrounds with varying levels of institutional resources. Moreover, a substantial proportion of the students enrolled in these state universities come from low-income and economically disadvantaged families who rely on free tuition and other scholarship privileges. The access to state- funded education provides a critical opportunity for overcoming financial barriers and attain social mobility which are one of the realities of Philippine public higher education. A stratified random sampling method is utilized to select respondents proportionately from various programs and year levels. This strategy ensured equal representation across key subgroups and reduced sampling bias (Creswell and Creswell, 2018). The sample size achieved the minimum suggested by Hair et al. (2021) for Partial Least Squares Structural Equation Modeling (PLS-SEM). Given the complexity of this study’s structural model, 1,026 student respondents were recruited to ensure adequate statistical power, validity, and generalizability.

### 2.3 Instrument

The study utilized a six-part questionnaire to measure students’ demographic profile, social capital, educational justice, institutional trust, student empowerment, and civic engagement, drawing from established theoretical frameworks such as Robert D. Putnam’s social capital theory, John Rawls’ theory of justice, and models of trust, empowerment, and civic participation. The assessment includes Factor Loading (FL), Cronbach’s Alpha, Composite Reliability, and Average Variance Extracted (AVE). Clearly, the range from 0.703 to 0.88 (FL) indicates that the indicators reliably measure each construct, demonstrating that the measured items are good representations of the constructs. Likewise, all values from both Cronbach’s alpha and Composite Reliability indicate strong internal consistency and minimal error. The HTMT values among the constructs range from 0.458 to 0.788, all of which fall well below the suggested threshold of 0.90 (Henseler et al., 2015). This ensures that each construct is empirically distinct from the others and does not exhibit multicollinearity. The Fornell-Larcker criterion results further confirmed that the square root of each construct’s AVE was higher than its inter-construct correlations, supporting construct distinctiveness. Overall, the results demonstrate that social capital, educational justice, institutional trust, student empowerment, and civic engagement are statistically reliable, conceptually distinct, and suitable for subsequent structural model analysis using PLS-SEM. The measurement model assessment results are shown in Tables 1, 2.

**Table 1:** Measurement model assessment results.

Constructs	Items	FL	Cronbach	CR	AVE
Civic engagement	CE1-CE6	0.717–0.872	0.892	0.918	0.615
Educational justice	EJ1-EJ7	0.703–0.846	0.908	0.927	0.645
Institutional trust	IT1-IT7	0.819–0.883	0.940	0.951	0.736
Social capital	SC1-SC7	0.744–0.799	0.886	0.911	0.593
Student empowerment	SE1-SE7	0.733–0.844	0.907	0.927	0.644

**Table 2:** Discriminant validity testing using Heterotrait-Monotrait Ratio and Fornell-Larcker criterion.

Constructs	Civic engagement	Educational justice	Institutional trust	Social capital	Student empowerment
Heterotrait-Monotrait Ratio (HTMT)					
Civic engagement					
Educational justice	0.458				
Institutional trust	0.573	0.788			
Social capital	0.643	0.537	0.597		
Student empowerment	0.740	0.676	0.699	0.687	
Fornell-Larcker criterion					
Civic engagement	0.807				
Educational justice	0.427	0.803			
Institutional trust	0.531	0.738	0.858		
Social capital	0.580	0.490	0.548	0.770	
Student empowerment	0.670	0.618	0.648	0.621	0.802

### 2.4 Data gathering procedure

The study was conducted and followed a three-stage approach from data collection to final interpretation. In the pre-data gathering stage, permissions were obtained from the university and college of interest, a structured questionnaire was developed and validated through expert reviews and pilot testing. Instructional sessions were arranged for coordinators or faculty representatives to acquaint them with the study’s purpose. When the questionnaire was ready, the researchers went into the field where it was administered face-to-face and online considering respondent’s availability and location. Coordination with college leaders and faculty and student leaders have facilitated smooth data collection. In the post-data gathering stage, the questionnaires were retrieved and organized before encoding and statistical analysis. The results then went beyond mere explanation to test hypotheses addressed in study objectives, forming the basis for conclusion, recommendations and the proposed development framework.

### 2.5 Data analysis

In order to obtain a correct and reliable result, the data collected in the study were systematically analysed using appropriate statistical tools. The statistical tools employed are frequency and percentage, mean and standard deviation, and partial least squares structural equation modelling (PLS-SEM). Frequency and Percentage, this statistical tool summarizes the respondents’ demographic profiles regarding age, sex, year level, and institution. In addition, Mean and Standard Deviation are also used to determine respondents’ levels of agreement with social capital, educational justice, institutional trust, student empowerment, and civic engagement. Finally, Partial Least Squares Structural Equation Modeling (PLS-SEM) was used to verify the constructs’ reliability and to check their validity, the model fit indices and indirect effects, as well as mediation.

## 3 Results

The demographic profile of the respondents by sex, age, year level and college affiliation is presented in Table 3. In terms of sex, the respondents were predominantly female (70.66%) compared to male (29.34% of the total sample). When assessed by age, most respondents belong to the age groups of 17–18 years old (40.66%) and 19–20 years old (33.33%), while those aged 21–22 years old (19.69%) and 23 years old above group (6.92%) comprise the smaller proportions. In terms of year level, most of the study’s participants are first-year students (53.22%), followed by second-year (18.81%), third-year (10.14) and fourth-year students (17.84). First-year students are represented relatively more in data collected as they were more numerous during the data gathering. This implies that a number of participants are in the fledgling stages of their academic paradigm; being fresh from academia means different attitudes, motivators and levels agreement with respect to the major variables. Lastly, most respondents came from the College of Education (55.17%), followed by the College of Technology (16.08%), the College of Engineering (14.72%), and the College of Management and Entrepreneurship (14.62%).

**Table 3:** Demographic profile of the respondents.

Variables	Profile	Responses	
		f	%
Sex	Male	301	29.34
	Female	725	70.66
Age	17–18 years old	411	40.06
	19–20 years old	342	33.33
	21–22 years old	202	19.69
	23 years old and above	71	6.92
Year level	First year	546	53.22
	Second year	193	18.81
	Third year	104	10.14
	Fourth year	183	17.84
College	College of education	566	55.17
	College of engineering	151	14.72
	College of management and entrepreneurship	150	14.62
	College of technology	165	16.08

### 3.1 Descriptive statistics and correlation analysis of the constructs

Descriptive statistics and correlation analysis of the key constructs in the study: social capital, educational justice, institutional trust, student empowerment, and civic engagement are presented in Table 4. Respondents tended to agree with the statements measuring these constructs, as indicated by mean scores ranging from 3.92 to 4.16. Civic engagement had the lowest mean (*M* = 3.92, SD = 0.78), but still showed high agreement; institutional trust had the highest mean (*M* = 4.16, SD = 0.72), indicating that respondents tended to agree to a much higher degree regarding institutional trust than civic participation. All constructs are positively and significantly associated with one another at the 0.01 level (2-tailed), as indicated by our correlation analysis, suggesting meaningful associations among the variables. In particular, institutional trust and justice in education had the largest correlation (*r* =0.725), indicating that when people perceive fairness and equity in education, they tend to have a high positive relationship with trust in institutional systems. Likewise, there were robust associations between student empowerment and social capital (*r* = 0.614) and civic engagement (*r* = 0.663), indicating that empowered students have greater access to social networks and civic engagement. These results suggest that the constructs are related and lay an initial foundation for investigating their relationships in future structural equation model analyses.

**Table 4:** Descriptive statistics and correlation analysis of the constructs.

Constructs	Mean	SD	1	2	3	4	5
Social capital	3.95	0.81	1				
Educational justice	3.96	0.80	0.478^**^	1			
Institutional trust	4.16	0.72	0.543^**^	0.725^**^	1		
Student empowerment	3.94	0.75	0.614^**^	0.608^**^	0.638^**^	1	
Civic engagement	3.92	0.78	0.569^**^	0.403^**^	0.519^**^	0.663^**^	1

**Correlation is significant at the 0.01 level (2-tailed).

### 3.2 Path analysis

Table 5 presents the results of the path analysis examining the relationships among social capital, educational justice, institutional trust, student empowerment, and civic engagement. Results indicate that social capital has a considerable effect on institutional trust (*β* = 0.245, *p* < 0.001), student empowerment (*β* = 0.418, *p* < 0.001), and civic engagement (*β* = 0.247, *p* < 0.001); meaning the higher the social ties between students at an institution leads to more trust students have in their institutions, more empowered, and better participation levels. Meanwhile, educational justice had a strong positive effect on institutional trust (*β* = 0.618, *p* < 0.001) and student empowerment as well (*β* = 0.413, *p* < 0.001). This shows that if students perceive the educational system fairly and equally, they place greater trust in their institution and become more socially active. Notably, educational justice had a sizeable, negative relationship with civic engagement (*β* = −0.116, *p* = 0.004), indicating that higher scores on perceptions of justice may, in fact, indirectly affect students’ civic engagement. Thus, social capital and educational justice explained institutional trust, student empowerment, and civic engagement in the expected manner, satisfying all of the hypotheses.

**Table 5:** Results of the path analysis.

Path	Beta (*β*)	*T* values	*p*-values	Result
H1: social capital → institutional trust	0.245	8.456	0.000	Supported
H2: social capital → student empowerment	0.418	12.302	0.000	Supported
H3: social capital → civic engagement	0.247	7.056	0.000	Supported
H4: educational justice → institutional trust	0.618	25.523	0.000	Supported
H5: educational justice → student empowerment	0.413	13.436	0.000	Supported
H6: educational justice → civic engagement	−0.116	2.879	0.004	Supported
H7: institutional trust → civic engagement	0.173	4.407	0.000	Supported
H8: student empowerment → civic engagement	0.477	11.691	0.000	Supported

Furthermore, the results indicate that institutional trust and student empowerment significantly influence students’ civic engagement. The result identified a positive and significant effect of institutional trust on civic engagement (*β* = 0.173, *p* < 0.001), indicating that students with higher levels of institutional trust are more engaged in civic and community activities. Civic engagement was most strongly influenced by student empowerment (*β* = 0.477, *p* < 0.001), whereby capable, confident, and involved students engage civically. The significant t-values, which are quite large across all proposed relationships, reaffirm their strength and significance. These findings foreground the important roles of social capital, educational justice, institutional trust, and student empowerment in shaping education systems’ constructions of students as civic actors. Overall, the structural model provides empirical support for the proposed relationships and emphasizes the importance of empowering students and fostering trust within educational institutions to promote civic engagement.

### 3.3 The mediating roles of institutional trust and student empowerment

The mediation path analysis and the indirect effects of social capital and educational justice on civic engagement through institutional trust and student empowerment are shown in Table 6. Results show that social capital (*β* = 0.043, *p* < 0.001) and educational justice (*β* = 0.107, *p* < 0.001) were significantly mediated by institutional trust, which in turn was significantly mediated by civic engagement. In the same way, student empowerment was a significant mediator of social capital on civic engagement (*β* = 0.199, *p* < 0.001) and educational justice to civic engagement (*β* = 0.197, *p* < 0.001). Among the mediation pathways, the pathway through student empowerment exhibited the largest indirect effect of social capital on civic engagement, followed by educational justice. The substantial t-values and *p*-values for all mediation paths confirm that institutional trust and student empowerment are potential mediational pathways through which social capital and educational justice promote students’ civic engagement. The results also substantiate each of the four mediation hypotheses presented, demonstrating the importance of trust and empowerment in strengthening students’ civic engagement.

**Table 6:** Results of the mediation path analysis.

Mediation path	Beta (*β*)	*T* values	*p*-values	Result
H9: social capital → institutional trust → civic engagement	0.043	4.041	0.000	Supported
H10: social capital → student empowerment → civic engagement	0.199	9.193	0.000	Supported
H11: educational justice → institutional trust → civic engagement	0.107	4.277	0.000	Supported
H12: educational justice → student empowerment → civic engagement	0.197	8.325	0.000	Supported

### 3.4 Final model of the study

**Figure 2:**
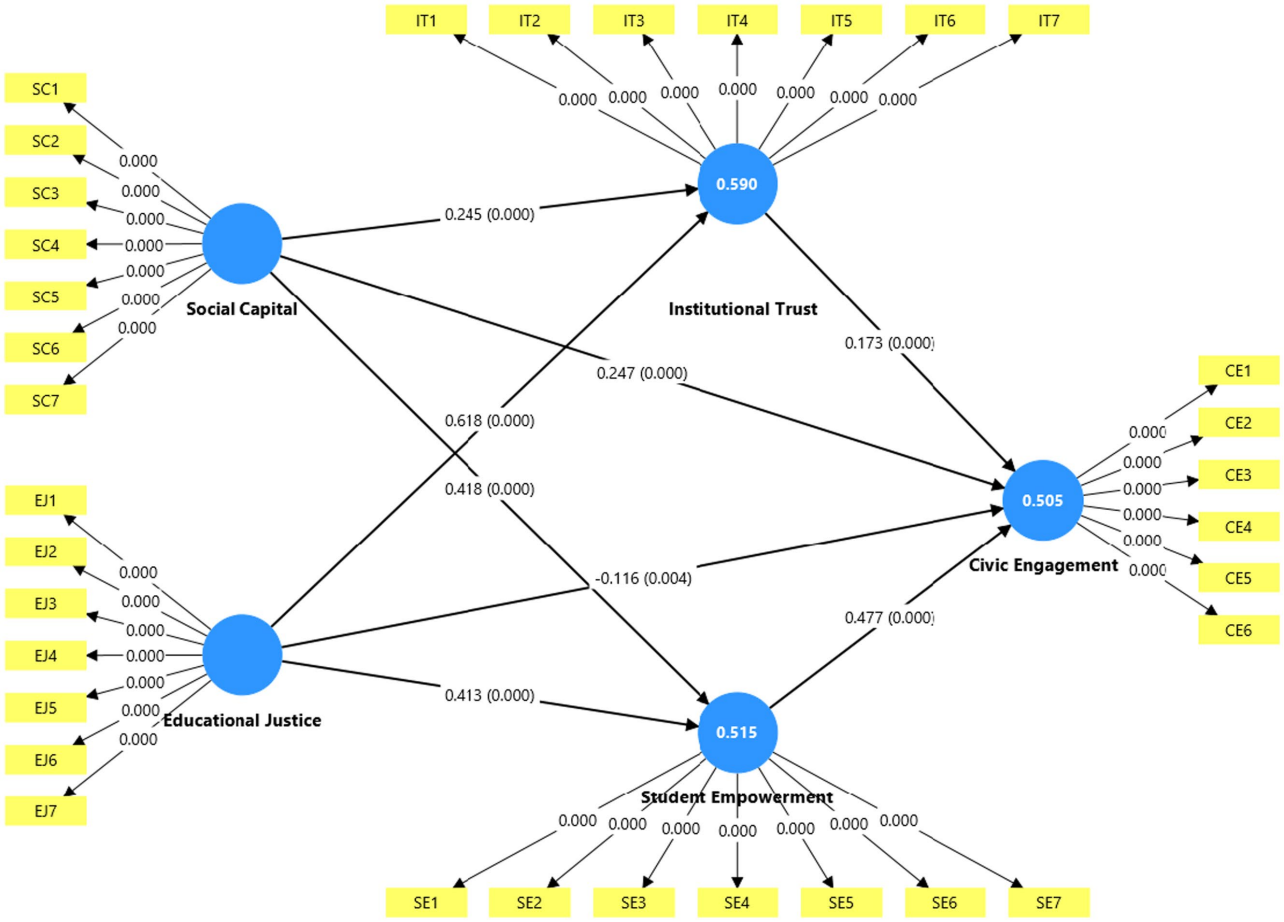
Final model of the study.

Figure 2 illustrates the final structural model of the study, showing the relationships among social capital, educational justice, institutional trust, student empowerment, and civic engagement. The model indicates that social capital and educational justice significantly influence institutional trust and student empowerment, which, in turn, contribute to civic engagement. Among the predictors, student empowerment has the strongest direct effect on civic engagement, underscoring the importance of empowering students to promote active participation in civic activities.

## 4 Discussion

Students generally show moderate to high agreement across the constructs measured in the study, indicating consistent perceptions. Institutional trust received the highest level of agreement (Mean = 4.16, SD = 0.72), suggesting that respondents strongly perceive the importance of trust in institutions. This kind of trust enhances students’ sense of loyalty and engagement, as they feel secure in an academic setting where their voices are heard and respected (Mekolle, 2024). While Civic Engagement had the lowest mean (3.92) and a slightly higher variability (SD = 0.78), indicating that while respondents still agree on its importance, there is slightly more variation in opinions. Moreover, the findings suggest that social ties shape students’ meaningful experiences, motivating them to actively engage with peers who support them holistically, as also supported by Merdiaty and Sulistiasih (2024). This also reflects one of the key aspects of bridging social capital, as highlighted by Quan-Haase et al. (2024), namely the respondents’ willingness to build connections beyond their close peer groups. Consequently, as educators model ethical conduct and good values that students can emulate, such practices can also strengthen trust and rapport between students and faculty (Kumar, 2024). Likely, Todorova and Tsanov (2024) found in their study a strong sense of ownership, in which students acknowledge their academic efforts and involvement in various activities, leading them to feel they positively adhere to and contribute to the fulfillment of their institution’s broader educational goals. Henceforth, to produce professional individuals who are more civically active and engaged, educators must not limit their ability to create accessible platforms. As such, teachers can also hone their civic attitudes toward social and political issues. Overall, the findings suggest that participants acknowledge the relevance of these constructs, with institutional trust being particularly emphasized.

Further, results show that all hypothesized relationships demonstrate statistically significant and supported, as evidenced by the *p*-values (*p* ≤ 0.004), which are all below the 0.05 significance level. Social capital has a significant effect on Institutional Trust (*β* = 0.245, *t* = 8.456), Student Empowerment (*β* = 0.418, *t* = 12.302), and Civic Engagement (*β* = 0.247, *t* = 7.056). This means that stronger social networks and relationships contribute to higher institutional trust, empower students, and develop civic participation. Likewise, social bonds create a sense of belonging, support, and access to diverse knowledge, which can significantly shape their views and opinions on fairness and transparency within the institution (Backhaus et al., 2020). Clearly, social capital also helps build trust and confidence in the students’ learning environment, as Gao et al. (2024) described. This also suggests that when they engage meaningfully in both academic and social interactions, they can develop a strong capacity to influence their friends and other significant individuals around them, as noted in the study by Gebregergis and Csukonyi (2024). The social relationships with their peer groups, as shared in the study by Li et al. (2022), not only boost collective action but also expose students to diverse situations related to societal issues and problems.

Similarly, educational justice has a strong positive impact on Institutional Trust (*β* = 0.618, *t* = 25.523) and student empowerment (*β* = 0.413, *t* = 13.436), suggesting that fair and equitable educational practices can foster students’ trust in their institutions and their sense of empowerment. This suggests that educational justice serves as a cornerstone for building institutional trust among students, likely making them feel that their institution operates with high integrity and transparency. In the study by Lomer and Lim (2022), these results emphasize that institutional efforts to ensure fair treatment, equitable access to resources, and impartial decision-making bolster students’ commitment to the institution. Henceforth, it can foster a supportive academic culture in which mutual respect and accountability are built and sustained. Predictably, educational justice plays a crucial role in nurturing empowered learners who are capable, confident, and actively invested in making their academic experiences in higher education relevant and meaningful (Trinidad and Leviste, 2021). Interestingly, Educational Justice has a negative but significant relationship with Civic Engagement (*β* = −0.116, *t* = 2.879), indicating a slight inverse association in this model. In this claim, educational justice within the school does not directly encourage students to be actively involved in civic-related activities. In practical terms, the influence of educational justice on civic behavior may be indirectly mediated by other factors, such as trust and empowerment (Imanipour, 2023), through which students are likely to channel their awareness of fairness into observable civic actions.

On the other hand, Institutional Trust significantly affects Civic Engagement (*β* = 0.173, *t* = 4.407), while Student Empowerment has the strongest direct influence on Civic Engagement (*β* = 0.477, *t* = 11.691). Consequently, the value of trust extends beyond the four walls of the classroom, as it may seem to. As highlighted by Trolian and Parker (2020), this is a critical strategy in the curriculum for educational institutions that aim to cultivate and produce socially responsible individuals who are actively engaged in civic life. Understandably, the study by Copeland et al. (2025) found that these individuals are not only aware of their rights and responsibilities but also motivated to take steps to effect change and make better decisions for the common good. Among the mediating predictors of civic engagement, student empowerment emerged as the strongest determinant, indicating its central mediating role in the model. Therefore, student empowerment serves as an instrumental bridge, transforming related constructs into aspiring civic values. Path analysis confirms that the conceptual model has good empirical support. Both social capital and educational justice are critical antecedents that fortify institutional trust and student empowerment, which in turn shape the nature of civic engagement. In this sense, the relationships among such patterns are profoundly enhanced by students’ civic-mindedness.

The mediation analysis results indicate that all proposed mediation paths are statistically significant and supported, as evidenced by *p*-values of 0.000, which are below the 0.05 significance level. The findings show that Institutional Trust significantly mediates the relationship between Social Capital and Civic Engagement (*β* = 0.043, *t* = 4.041) and between Educational Justice and Civic Engagement (*β* = 0.107, *t* = 4.277), suggesting that trust in institutions strengthens the influence of social capital and educational justice on civic engagement. Indeed, students with supportive social networks are more likely to trust their institutions, which in turn encourages greater participation in civic activities. Thus, institutional trust positively mediated the relationships between educational justice and civic engagement. This also indicates that respondents’ perceptions of fairness and equity influence their civic involvement, as exemplified by Mitchell et al. (2018). On a positive note, trust in academic processes provides a sense of security and commitment, encouraging students to participate in civic activities (Platz, 2021). Ideally, while educational justice relates to the foundational concepts of fairness, it is the mediating influence coming from institutional trust (*β* = 0.107, *t* = 4.277, *p* = 0.000) that translates these concepts into civic attitudes, showing the indirect but significant role of trust in developing the student’s full potential regarding their engagement in societal concerns. These findings highlight the pivotal role of institutional trust as a bridge connecting equitable educational environments and robust social networks to students’ civic engagement.

Student Empowerment also serves as an important mediator, as shown in the relationship between Social Capital and Civic Engagement (*β* = 0.199, *t* = 9.193) and between Educational Justice and Civic Engagement (*β* = 0.197, *t* = 8.325). Students who are embedded in strong social relationships have heightened their sense of agency and belonging. Research by Xin (2022) further emphasized that students’ social support significantly predicts civic engagement. Social capital alone may not fully explain civic behaviors, but self-empowerment within the academic setting can be translated into meaningful civic engagement. Accordingly, as Van Matre (2025) noted, varied pedagogical methods can empower students and foster civic traits by fostering social capital in classrooms that enhance students’ participation in civic activities. This also highlights the critical role in creating supportive environments in higher educational institutions. A supportive environment can encourage civic skills, which are essential foundations for responsible citizenship. Truly, students feel empowered when they observe fairness in their learning environment (Killen and Rutland, 2022). When the institution acknowledges their presence, the students then understand that their voices matter. Perhaps a fair environment lies at the core of civic engagement, but it is through empowerment that these perceptions are translated into tangible civic actions. Indeed, as noted by Skarmeas et al. (2020), a truly empowered individual increases their involvement in civic affairs. Notably, the mediation effect through Student Empowerment is stronger than through Institutional Trust, indicating that empowering students plays a more substantial role in translating social capital and educational justice into active civic engagement. Overall, these results highlight the importance of both institutional trust and student empowerment as key mechanisms that enhance the impact of social capital and educational justice on students’ civic life.

The findings of the observed constructs may also be understood and influenced, considering the respondent’s contextual characteristics. Unlike many studies conducted in highly resourced universities, the respondents were enrolled in a state-funded institution where most students come from financially disadvantaged backgrounds. To pursue their academic goals, they rely heavily on the varied academic opportunities provided within their academic environment. As beneficiaries, students have placed greater value on institutional support and trust, opportunities for participation, and fairness as these factors directly influence their future aspirations in the university. Engagement within the university community has become particularly important for building social networks and fostering self-confidence. Therefore, the positive relationships observed among the constructs suggest that equitable and supportive learning environments are instrumental in shaping students’ civic skills. In like manner, the findings have also revealed the critical role of educational institutions in cultivating socially responsible and civically engaged students.

## 5 Conclusion and recommendations

The study concludes that the significant influence of social capital and educational justice, acting indirectly, promotes civic engagement in higher education institutions. Moreover, student empowerment and institutional trust played an important mediating role in actively cultivating civic behaviors and attitudes. The mediation path analysis also indicated that student empowerment is the most influential mediator of students’ civic participation. Additionally, the findings confirmed that when students experience inclusivity and fair treatment, they feel more empowered. In this regard, they are more likely to become civically minded individuals. Furthermore, the results underscore the importance of implementing concrete institutional practices that can strengthen both structural (social capital and educational justice) and psychological (trust and empowerment) conditions within higher education institutions to foster an effective and active civic life among students. More inclusive student development and leadership programs, as well as community engagement initiatives, can be recommended to allow students to practice institutional and civic processes.

## Data Availability

The original contributions presented in the study are included in the article/Supplementary material, further inquiries can be directed to the corresponding author.
