# Supplementary material for: Social capital and educational justice as predictors of civic engagement in higher education: the mediating roles of institutional trust and student empowerment
Source: Front Sociol. 2026 Jul 9;11:1869260. doi: 10.3389/fsoc.2026.1869260 (PMC13391324; doi:10.3389/fsoc.2026.1869260)
Supplement: Supplementary file 1 [file Supplementary_file_1.docx]

**APPENDIX A**

**Scoring Procedure**

Table 1 presents the interpretation framework for the five-point Likert scale used in the study, where each numeric range corresponds to a level of agreement or disagreement. Scores between 1.00 and 1.79 indicated strong disagreement with a statement, while scores between 1.80 and 2.59 reflect general disagreement. Scores between 2.60 and 3.39 indicated a neutral stance, neither agreement nor disagreement. Scores ranging from 3.40 to 4.19 suggested agreement, and scores from 4.20 to 5.00 signify strong agreement. This scale provided a standardized method for quantifying respondents’ attitudes, perceptions, or opinions toward various survey items.

**Five-point Likert Scale**

| **Scale** | **Range** | **Interpretation** |
| --- | --- | --- |
| 1 | 1.00-1.79 | Strongly Disagree |
| 2 | 1.80-2.59 | Disagree |
| 3 | 2.60-3.39 | Neutral |
| 4 | 3.40-4.19 | Agree |
| 5 | 4.20-5.00 | Strongly Agree |
