# Supplementary material for: Social capital and educational justice as predictors of civic engagement in higher education: the mediating roles of institutional trust and student empowerment
Source: Front Sociol. 2026 Jul 9;11:1869260. doi: 10.3389/fsoc.2026.1869260 (PMC13391324; doi:10.3389/fsoc.2026.1869260)
Supplement: Supplementary file 2 [file Supplementary_file_2.docx]

**APPENDIX B**

| Constructs | Code | Items | References |
| --- | --- | --- | --- |
| Social Capital | SC1  SC2  SC3  SC4  SC5  SC6  SC7 | I have strong relationships with classmates and peers in my academic program.  I can rely on my close friends in school for emotional and academic support.  I share similar values and goals with my close circle of peers.  I trust my peers to support me when I face challenges.  I interact positively with students from other academic programs.  I participate in events that allow me to connect with students from different backgrounds.  I am open to collaborating with people whose views differ from mine. | Paiva et al. (2014); Grootaert et al. (2004); Pishghdam (2011) |
| Educational Justice | EJ1  EJ2  EJ3  EJ4  EJ5  EJ6  EJ7 | Academic resources are distributed fairly among all students.  Scholarships and financial aid are accessible to those who need them most.  The allocation of facilities supports equal learning opportunities for everyone.  Policies and rules in the school are applied consistently to all students.  Students have a voice in decision-making processes that affect them.  The grievance process in the school is fair and transparent.  Faculty members treat students with dignity and respect. | Karakoc & Sakiz (2021); Zhang et al. (2018); Rezai (2022) |
| Institutional Trust | IT1  IT2  IT3  IT4  IT5  IT6  IT7 | I believe my institution acts in the best interest of its students.  I trust the school to handle my personal information responsibly.  I believe the administration is competent in managing the school.  I have confidence in the institution’s commitment to quality education.  I believe my institution is transparent in its policies and decisions.  Faculty members act in the best interest of students.  My university upholds its commitments to students. | Deniz et al. & Erdener (2023); Carvalho & de Oliveira Mota (2010); Dziminska et al. (2018) |
| Student Empowerment | SE1  SE2  SE3  SE4  SE5  SE6  SE7 | I believe my role as a student contributes meaningfully to my institution’s goals.  I feel my opinions in school activities have value.  I feel confident in expressing my opinions in class.  I am confident in my ability to accomplish academic tasks successfully.  I can handle academic challenges effectively.  I can choose activities that align with my interests and goals.  I feel empowered to suggest changes in school policies or programs. | Kirk et al. (2016); Terkamo- Moisio (2022); Fei et al. (2025) |
| Civic Engagement | CE1  CE2  CE3  CE4  CE5  CE6  CE7 | I participate in student government elections.  I attend forums or discussions about social and political issues.  I take part in activities that promote social responsibility.  I contribute to projects that benefit the community.  I value helping others in my community.  I am interested in participating in civic or political activities.  I actively participate in community service and local initiatives that address social or environmental issues. | Park (2025) & Hong (2025); Doolittle & Faul (2013) |
